# Crystal Structure and Size-Dependent Neutralization Properties of HK20, a Human Monoclonal Antibody Binding to the Highly Conserved Heptad Repeat 1 of gp41

**DOI:** 10.1371/journal.ppat.1001195

**Published:** 2010-11-18

**Authors:** Charles Sabin, Davide Corti, Victor Buzon, Mike S. Seaman, David Lutje Hulsik, Andreas Hinz, Fabrizia Vanzetta, Gloria Agatic, Chiara Silacci, Lara Mainetti, Gabriella Scarlatti, Federica Sallusto, Robin Weiss, Antonio Lanzavecchia, Winfried Weissenhorn

**Affiliations:** 1 Unit of Virus Host Cell Interactions (UVHCI) UMI 3265, Université Joseph Fourier-EMBL-CNRS, Grenoble, France; 2 Institute for Research in Biomedicine, Bellinzona, Switzerland; 3 Division of Viral Pathogenesis, Beth Israel Deaconess Medical Center, Boston, Massachusetts, United States of America; 4 Humabs SAGL, Bellinzona, Switzerland; 5 Viral Evolution and Transmission Unit, Division of Immunology, Transplant and Infectious Diseases, San Raffaele Scientific Institute, Milan, Italy; 6 Division of Infection and Immunity, University College London, London, United Kingdom; 7 Institute of Microbiology, Swiss Federal Institute of Technology, Zurich, Switzerland; University of Zurich, Switzerland

## Abstract

The human monoclonal antibody (mAb) HK20 neutralizes a broad spectrum of primary HIV-1 isolates by targeting the highly conserved heptad repeat 1 (HR1) of gp41, which is transiently exposed during HIV-1 entry. Here we present the crystal structure of the HK20 Fab in complex with a gp41 mimetic 5-Helix at 2.3 Å resolution. HK20 employs its heavy chain CDR H2 and H3 loops to bind into a conserved hydrophobic HR1 pocket that is occupied by HR2 residues in the gp41 post fusion conformation. Compared to the previously described HR1-specific mAb D5, HK20 approaches its epitope with a different angle which might favor epitope access and thus contribute to its higher neutralization breadth and potency. Comparison of the neutralization activities of HK20 IgG, Fab and scFv employing both single cycle and multiple cycle neutralization assays revealed much higher potencies for the smaller Fab and scFv over IgG, implying that the target site is difficult to access for complete antibodies. Nevertheless, two thirds of sera from HIV-1 infected individuals contain significant titers of HK20-inhibiting antibodies. The breadth of neutralization of primary isolates across all clades, the higher potencies for C-clade viruses and the targeting of a distinct site as compared to the fusion inhibitor T-20 demonstrate the potential of HK20 scFv as a therapeutic tool.

## Author Summary

The HIV-1 envelope glycoprotein composed of the receptor binding subunit gp120 and the fusion protein gp41 is the prime target for neutralizing antibodies. Receptor binding induces a conformational change in gp41 that transiently exposes the conserved heptad repeat 1 (HR1) region. We have previously isolated the human HR1-specific mAb HK20 and provide now the structural basis for epitope recognition. HK20 employs mainly its CDR H2 and H3 for binding similar to HR1 binding of mAb D5. We demonstrate that HK20 and D5 bind HR1 with similar affinities; however, HK20 has a broader neutralization breadth than D5, which might be due to the differences in their approach angles of epitope recognition. Competition analyses of 33 sera from HIV-1 infected individuals reveal significant titers of HK20-inhibiting antibodies in 20 cases, confirming the immunogenicity of the epitope. We demonstrate further that HK20 IgG have limited neutralization breadth and potency while smaller HK20 Fabs and scFv reveal a broad cross clade neutralization breadth. This suggests that the accessibility of the HR1 epitope limits the value of HR1 mAbs for infection prevention, but highlights the importance of smaller versions such Fabs or scFv to combat infection alone or in synergistic approaches with other antivirals.

## Introduction

The HIV-1 envelope (Env) glycoprotein is the main target for neutralizing antibodies. Thus a successful HIV-1 vaccine must induce broadly cross-clade neutralizing antibodies as an essential correlate of protection against infection [1]. The HIV-1 genome and especially its *env* gene is highly variable between and within clades [2], which is partly responsible for the difficulty in developing a suitable vaccine candidate [3], [4]. Consequently, the search for conserved targets is the basis of current attempts to develop an effective HIV-1 vaccine.

Trimeric Env is composed of the receptor binding domain gp120, which is non-covalently associated with the membrane-anchored fusion protein gp41. Infection of target cells is initiated by the attachment of Env to the CD4 receptor [5], [6], which triggers conformational changes that expose the hypervariable loop 3 (V3) [7], thus priming it for co-receptor CCR5 or CXCR4 interaction [8], [9]. Together CD4 and co-receptor interactions are thought to induce conformational changes in the fusion protein subunit resulting in exposure and subsequent insertion of the fusion peptide into the target cell membrane which produce the fusion intermediate pre-hairpin structure that bridges viral and cellular membranes [10], [11]. During this process heptad repeat regions 1 (HR1) and 2 (HR2) are transiently exposed [12] permitting interaction with peptide inhibitors of fusion such as T-20 [13], [14]. Subsequent refolding of the pre-hairpin structure into the post-fusion conformation [15], [16], [17], [18] leads to the apposition of viral and cellular membranes catalyzing membrane fusion [19].

The fusion-intermediate conformation of gp41 is an attractive target for neutralizing antibodies due to its relative high sequence conservation. Broadly cross-clade neutralizing antibodies 2F5, 4E10 and Z13 target the membrane proximal region most likely during epitope exposure in the fusion-intermediate pre-hairpin conformation [20], [21], [22]. A number of monoclonal antibodies directed against HR1 exposed in the pre-hairpin conformation of gp41 have been isolated from phage display libraries, which show variable neutralization profiles depending on the neutralization assays used. MAb D5 was isolated from a naïve human library [23] and MAb DN9 from a Fab library generated from bone marrow RNA from an HIV-1 infected individual [24], while the rabbit single chain mAb 8K8 was derived from a phage library [24] prepared from rabbits immunized with a gp41 HR1 mimetic [25]. Several HR1-specific Fabs were also isolated from a human non-immune phage library [26], [27] and Fab 3674 was *in vitro* matured [28]. Notably, immunization strategies employing HR1 peptide mimetics led to the generation of a polyclonal antibody response capable of neutralizing Tier 1 primary isolates [29].

The crystal structure of the D5 Fab in complex with the gp41 mimetic 5-Helix [30] reveals that D5 binds orthogonal to the axis of the HR1 trimer. The principal interaction site is a conserved hydrophobic pocket on gp41 [31] that is the target for HR2 [15], [16], D-peptides and various peptide mimetics [25], [32], [33], demonstrating that D5 binding blocks the transition into the six-helix conformation required for entry [31].

We previously reported the isolation of the gp41-specific antibody HK20 from immortalized memory B cells of an HIV-1 infected individual, which targets the conserved hydrophobic pocket in gp41 HR1 [34]. HK20 has a considerable breadth of neutralization on isolates from multiple clades. However the neutralizing potency was low and target cell dependent [34].

Here we present the crystal structure of the HK20 Fab in complex with gp41 5-Helix as well as a detailed characterization of HK20 neutralizing properties. The structure reveals that HK20 occupies a conserved hydrophobic pocket within the HR1 triple stranded coiled coil, indicating that HK20 binding inhibits membrane fusion by interfering with HR2 refolding into the six helix bundle post fusion conformation. Although HK20 and D5 occupy the same site on HR1, their angle of interaction vary substantially, which might account for the differences in neutralization potencies. Comparison of the neutralization activities in different assays reveals a much higher potency for smaller HK20 Fab and scFv over IgG, suggesting steric as well as temporal constraints. HK20 scFv neutralize all pseudoviruses tested as well as infectious viruses by targeting a conserved site that is not affected by T-20 resistance mutations.

## Results

### Structure of the HK20 Fab in complex with 5-Helix

**Figure 1:**
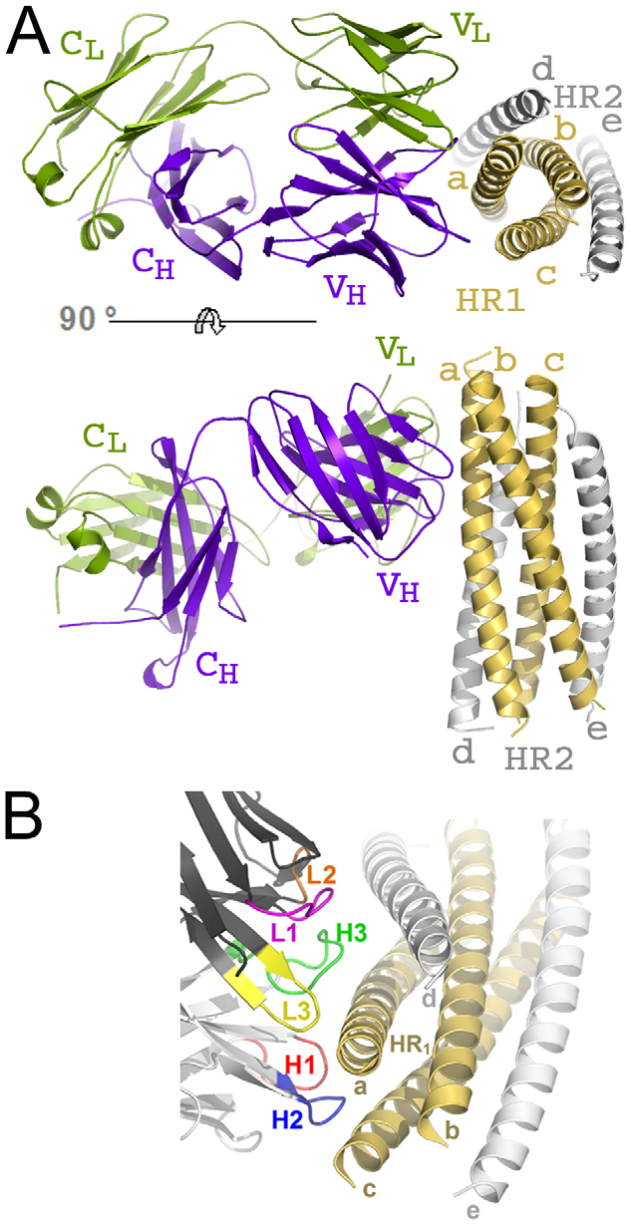
HK20 Fab binds to a conserved epitope on gp41 HR1. (**A**) Ribbon diagram of the HK20 Fab (heavy chain in blue and light chain in green) approaching its epitope on gp41 (HR1 in yellow and HR2 in grey) looking down the three-fold axis of gp41 (upper panel). The lower panel shows the side view of the complex and shows that the Fab complex approaches 5-Helix with an approximate angle of 60°. (**B**) Close-up of the HK20 interaction with 5-Helix. The complementarity determining regions are coloured differently and labelled H (heavy chain) and L (light chain).

**Figure 2:**
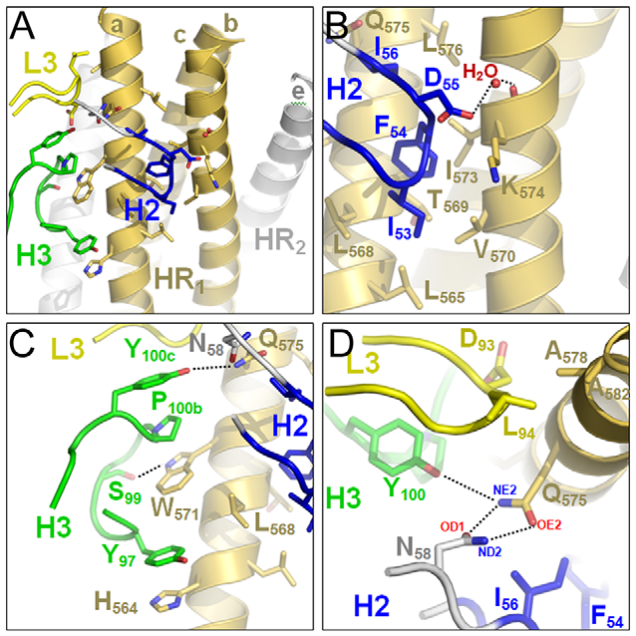
Close-up of the HK20-5-Helix interactions. (**A**) Only CDR H2 (blue), H3 (green) and L3 (yellow) contact two HR1 helices of 5-Helix. (B) Close-up of the hydrophobic and polar interactions mediated by CDR H2. (**C**) Close up of CDR H3 interactions highlighting the sandwich structure produced by residues H564, Y97, W571, P100b and Y100c. (**D**) Close-up of the polar network formed by CDR H2 N58, H3 Y100c and gp41 HR1 chain A Q575.

**Figure 3:**
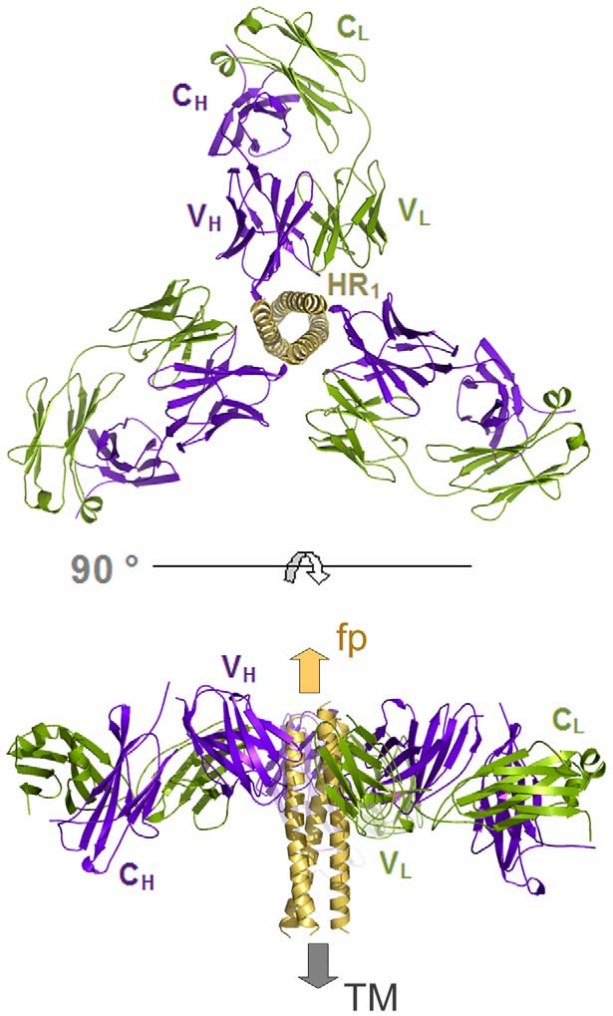
The gp41 prehairpin structure can bind three HK20 mAbs simultaneously. Molecular model demonstrating the docking of three HK20 Fabs onto three HR epitopes formed by three HR1 helices (Figure 1A; a-c, c-b and a-b). Note that no molecular clashes occur upon binding of all three Fabs. The upper panel shows the complex as viewed down the gp41 three-fold axis and the lower panel shows the complex in a side view indicating the orientations of the membrane anchors. The arrows show the directions of the viral membrane and of the fusion peptide that will be inserted into the target cell membrane.

**Figure 4:**
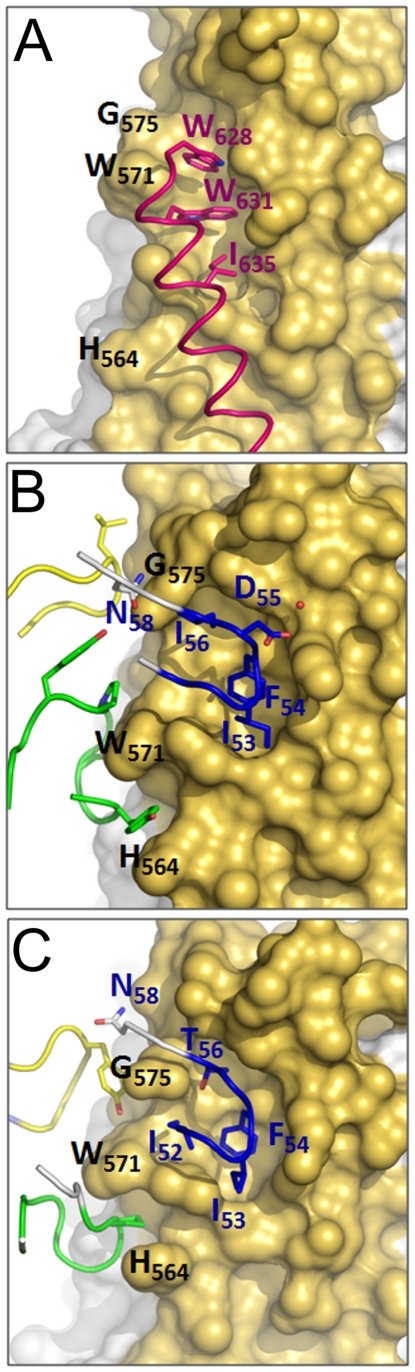
HK20 occupies a hydrophobic pocket on HR1, which is filled by HR2 in the six helix bundle post fusion conformation. (**A**) Close-up of the six helix bundle conformation of the HK20 epitope. The HR1 trimer is shown as a molecular surface (yellow) and the HR2 helix pointing residues Trp626, Trp631 and Ile635 into the pocket is shown as coil motif. (**B**) Close up of the HK20 CDR H2 pointing into the hydrophobic pocket together with the main structural principles of CDR H3 and L3 interactions. (**C**) Close-up of the D5 interaction sites reveals similar structural principles as shown for HK20 (see B).

The crystal structure of HK20-5-Helix complex was solved by molecular replacement and refined to a resolution of 2.3 Å (**Table 1**). The variable domains (V_H_ and V_L_) of the Fab approach the epitope in an ∼60° angle with respect to the 5-Helix trimer axis (**Figure 1A**). HK20 employs the complementarity determining regions (CDR) H2, H3 and L3 to contact two adjacent HR1 helices (**Figure 1B**). The tip of CDR H2 is a central determinant of interaction (**Figure 2A**) and positions Ile53 and Phe54 into a hydrophobic HR1 pocket (lined by HR1 chain a Leu565, Leu568, Thr569 and HR1 chain c Val570* and Ile573*) (**Figure 2B**). Furthermore CDR H2 Asp55 makes a water mediated contact to the carbonyl of Lys574 followed by a hydrophobic contact of Ile56. CDR H3 contacts HR1 chain a. The aromatic ring of Tyr97 is within π stacking distance to His564 and its orientation is supported by a hydrogen bond to the carbonyl of CDR H1 Arg31. The carbonyl of CDR H3 Ser99 hydrogen bonds to Trp571 NE1, whose aromatic ring forms a hydrophobic sandwich with the ring structure of Pro100b, itself stacked by Tyr100c (**Figure 2C**). Tyr100c also participates in a network of hydrogen bonds by contacting Gln575, which makes a double dent hydrogen bond to CDR H2 Asn58 (**Figure 2D**). The binding contribution of the light chain is minor and restricted to hydrophobic contacts by Asp93 and Leu94 to Ala578 and Ala582 of HR1 (**Figure 2D**). A water molecule coordinated by CDR H3 Ser98 and the carbonyl of Tyr97 contacts HR2 His 643. Docking demonstrates that three Fabs or antibodies could bind simultaneously to all three epitopes once they become exposed during the fusion reaction (**Figure 3**). The crystal structure reveals that the tip of HK20 CDR H2 occupies the hydrophobic pocket on HR1 that, in the post-fusion conformation, is filled by HR2 residues Trp626, Trp631 and Ile635 (**Figure 4A**) [15], [16]. In addition, the side chain of Trp571 rotates by ∼90° to accommodate binding to CDR H3 (**Figures 4A, B** **and** **2C**). Thus the HK20 interaction blocks entry by preventing the folding of HR2 onto HR1 which is required to catalyze fusion of viral and cellular membranes in order to establish infection [35].

**Table 1:** Data collection and refinement statistics.

Data collection	
Space group	P2_1_
Cell dimensions	
*a*, *b*, *c* (Å), β (°)	75.95, 62.71, 76.61; 97.70°
Wavelength (Å)	0.9765
Resolution (Å)	45.00-2.30 (2.42 – 2.30)^a^
Mesured reflections	95264
Unique reflections	28254
*R*_merge_	0.12 (0.35)
*I* / σ*I*	4.0 (2.1)
Completeness (%)	93.30 (89.20)
Redundancy	3.2 (2.4)
Wilson B factor (Å^2^)	32.00

Refinement	
Resolution (Å)	37.60 - 2.30
*R*_cryst_ (N° reflections)	0.239 (28254)
*R*_free_ (N° reflections)	0.279 (1509)
No. atoms	
Protein	4960
Water	118
*B*-factors (Å^2^)	35.97
R.m.s deviations	
Bond lengths (Å)	0.009
Bond angles (°)	1.183

a Number in parentheses refer to the highest resolution shell.

### Comparison of the HK20 and D5 interaction with 5-Helix

Fab D5, previously isolated from a human naive phage display library, uses similar structural principles for interaction with HR1 [31]. Its CDR H2 tip reaches into the HR1 pocket employing Ile53 and Phe54 for interaction (**Figure 4C**) with Phe54 occupying the identical position of HK20 CDR H2 Phe54 (**Figure 4B**). In contrast, D5 residue Gly55 replaces the important HK20 CDR H2 residue Asp55. D5 binding is supported by a shorter CDR H3 compared to HK20 (**Figure S1 in Supporting Information S1**), whereas D5 CDR H3 Pro97 is sandwiched between HR1 chain A residues His564 and Trp571 (**Figure 4C**). Other D5 contacts are provided by CDR L3 and by two interactions with HR2 residues, including a water mediated contact to HR2 His643 [31] as observed in case of HK20 interaction. Although HK20 and D5 VH domains are encoded by very similar germline genes, namely VH1-69*5 and VH1-69*1, HK20 shows four amino acid somatic mutations in CDR H2, while D5 uses the germline sequence of CDR H2 for interaction (**Figure S1 in Supporting Information S1**). The second important contribution of interaction comes from the CDR H3 region, which is completely different in both antibodies since it is encoded by D6-6*01 and J3*02 gene segments (HK20) and D1-14*1 and J4*02 gene segments (D5). Both antibodies employ different light chain gene segments (HK20 V_L_, V33*1 and J4*02; D5 V_L_, V5*01 and J4*1).

**Figure 5:**
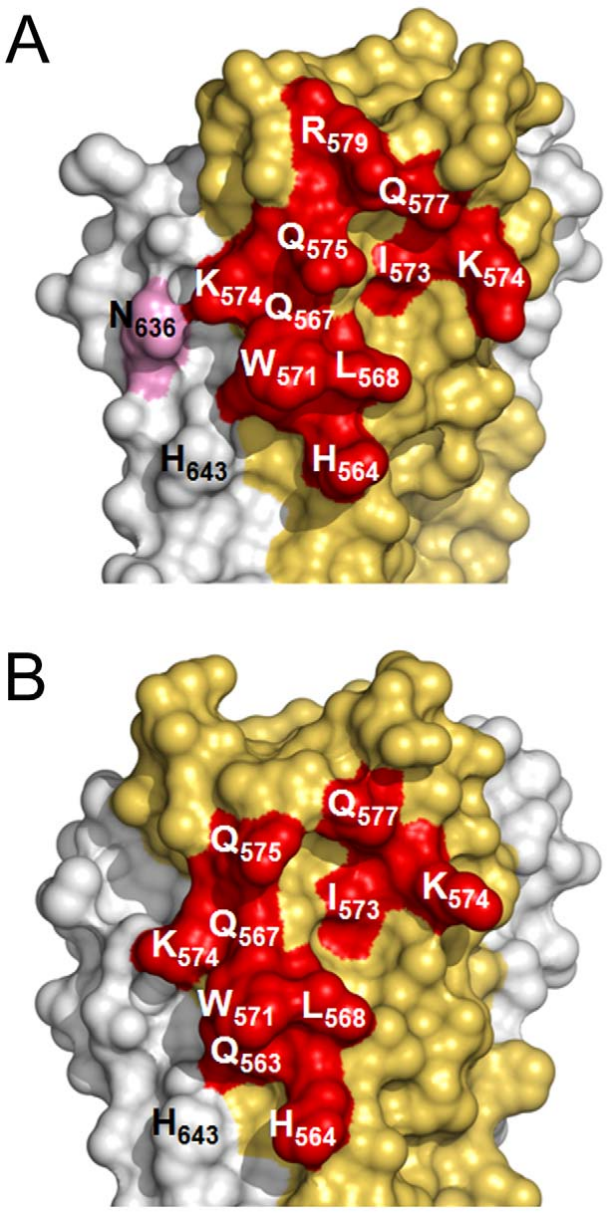
Surface representation of the HK20 and D5 footprints on 5-Helix. Interface area of HK20 (**A**) and of D5 (**B**). The HR1 surface is shown in yellow, the HR2 surface in grey and residues making direct contacts with HR1 are marked in red and HR2 in pink. Both HK20 and D5 interact indirectly with HR2 His643 via water-mediated hydrogen bonds.

**Figure 6:**
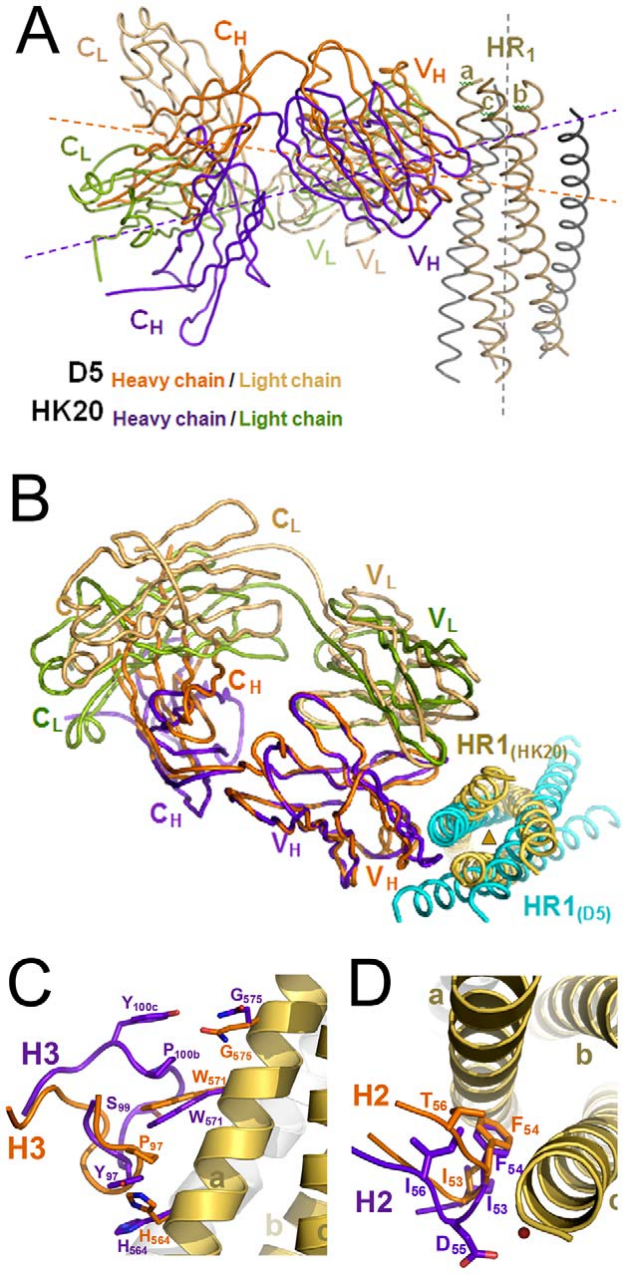
Cα super positioning of the HK20-5-Helix and the D5-5-Helix complexes reveals differences in epitope approach angles. (**A**) Cα super positioning of the 5-Helix HR1 helices A and C reveals differences in the approach angles of both complexes. While D5 binds in an orthogonal way to its HR1 epitope, HK20 approaches the epitope in an ∼60° angle. (**B**) The difference in binding angle is also supported by the Cα super positioning of V_H_ and HR1 chains a and c. (**C**) Close-up of the super positioning of HK20 and D5 CDR H3 loops demonstrating their differences in interaction with the HR1 helix. (**D**) Close-up of the super positioning of HK20 and D5 CDR H2 loops highlights the central role of Phe54 contacting two HR1 helices.

In spite of structural differences described above, both mAbs show a similar interaction footprint on the 5-Helix structure, with HK20 and D5 occupying a surface of 1061 Å^2^ and 1156 Å^2^, respectively (**Figures 5A, B**). Notably, Cα super positioning of both complexes reveals that HK20 approaches its epitope on HR1 in a different angle than D5. While D5 binds almost perpendicular to the 5-Helix trimer axis [31], HK20 approaches the epitope in a ∼60° angle between the 5-Helix trimer axis and the HK20 major axis (**Figure 6A**). This difference in interaction is more visible when the Cα atoms of the Fabs are super positioned, which reveals the dramatic change in HR1 trimer axis orientation between the two complexes (**Figure 6B**). The close up of the Cα super positioning highlights the distinct and common features of interaction of CDR H3 (**Figure 6C**) and H2 (**Figure 6D**) indicating that the same epitope can be targeted by different approach angles.

### Binding kinetics and role of somatic mutation

Surface plasmon resonance measurements showed an affinity constant (K_D_) of 3.3 nM for HK20 and 3.1 nM for D5 IgG binding to a gp41 HR1 mimetic. Both HK20 and D5 show similar association rates of k_a_ = 2.20±0.015 10^5^ M^−1^ s^−1^ and k_a_ = 3.35±0.022 10^5^ M^−1^ s^−1^ and similar dissociation rates of k_d_ = 7.27±1.05 10^−4^ s^−1^ and k_d_ = 10.3±0.089 10^−4^ s^−1^, respectively, suggesting that the minor difference in affinity does not explain the differences in neutralization as outlined below.

The contribution of somatic mutations was investigated by comparing, employing a 5-Helix-based ELISA, HK20, D5 and HK20 variants in which the VH and/or VL were reverted to the germline configuration. HK20 and D5 showed comparable EC50 values (0.1 µg/ml), while the HK20 variants in which the heavy chain alone or both heavy and light chains were reverted to germline showed approximately 50-fold lower binding (EC50 6.2 and 4.8 µg/ml). In contrast the germline reversion of VL alone had no measurable effect on binding using the ELISA assay (**Figure S2A in Supporting Information S1**). These binding results were also confirmed by the lack of neutralizing activity by the germline HK20 version or by the HK20 variant in which the light chain was reverted to germline when tested in parallel with HK20 in the HOS.CD4-R5 target cell assay against clade A and C HIV-1 isolates (**Figure S2B and C in Supporting Information S1**). These results are consistent with structural data described above and demonstrate that somatic mutations in the light chain do not contribute significantly to binding, while those in the heavy chain represent the positive contribution of somatic hyper-mutations to the affinity maturation of mAb HK20.

### Detection and quantification of HK20-like antibodies in patient sera

**Figure 7:**
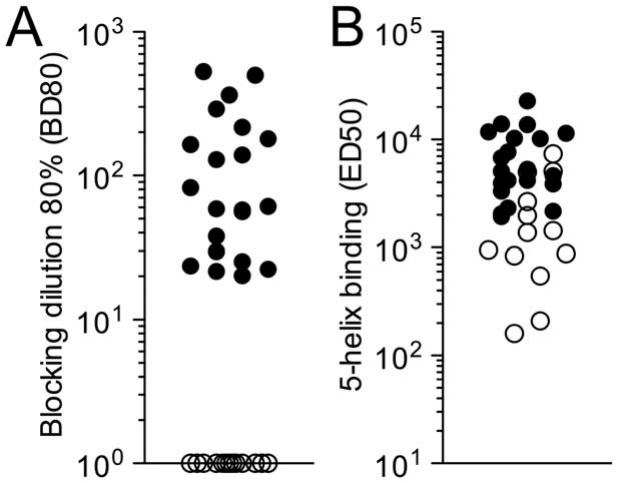
Detection of HK20-like antibodies in patient sera. (**A**) ELISA plates were coated with 5-Helix and incubated with serial plasma dilutions, followed by biotinylated HK20 mAb and by enzyme-conjugated streptavidin. Shown is the reciprocal plasma dilution that blocks 80% binding (BD80) of HK20 mAb. BD80 values <20 (empty circles) were scored as negative. Each symbol represents a different individual. (**B**) Total serum IgG binding to 5-Helix was determined by ELISA. Reciprocal EC50 values are shown. Empty circles correspond to plasma samples with BD80 values <20.

HK20 was an affinity selected antibody isolated from the memory B cell repertoire of an HIV-1 infected individual. To establish whether similar antibodies would be generally produced in the context of the immune response to HIV-1 infection we developed a competitive assay suitable for the detection and quantification of HK20-like antibodies in patients' sera. Serial dilutions were tested for their capacity to inhibit binding of biotinylated HK20 to immobilized 5-Helix. Twenty out of 33 sera from HIV-1 infected individuals showed significant titers of HK20-inhibiting antibodies, while the remaining did not show significant inhibition, in spite of variable binding to 5-Helix (**Figure 7A and B**). We conclude that the HK20-footprint is targeted by antibodies in a significant fraction of HIV-infected patients.

### Comparison of HK20 and D5 neutralizing activity

**Figure 8:**
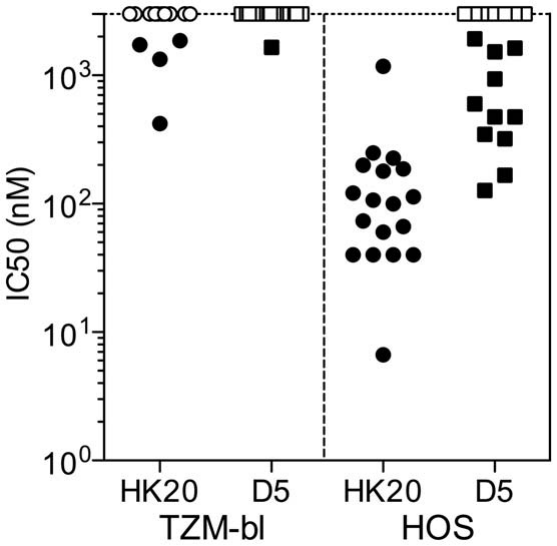
Comparison of HK20 IgG and D5 IgG in TZM-bl and HOS-based neutralization assays. HK20 (circles) and D5 (squares) were tested for their capacity to neutralize the same 18 HIV-1 pseudoviruses representing 6 different clades in TZM-bl and HOS-cell based assays. Shown are nM IC50 values, (empty circles or squares, IC50 >2000 nM (>300 µg/ml).

HK20 and D5 IgG were compared for their capacity to neutralize a panel of 18 HIV-1 Tier-1 and Tier-2 isolates spanning 6 clades. The assays were performed using either TZM-b1 or HOS.CD4-R5 as target cells. In the TZM-b1-based assay HK20 neutralized 4 out of 18 HIV-1 isolates with IC50 values below 300 µg/ml (<2000 nM), while D5 neutralized only one Tier 1 virus (**Figure 8** **and Table S1 in Supporting Information S1**). In contrast in the HOS-based assay, HK20 neutralized all 18 isolates tested with IC50 values ranging from 7 to 1173 nM, while D5 neutralized only 11 out of 18 isolates with IC50 values ranging from 126 to 1930 nM (**Figure 8** **and Table S1 in Supporting Information S1**). Thus, in spite of a similar molecular interaction with the target epitope, HK20 shows a higher potency and breadth of neutralization as compared to D5.

### Size dependent neutralization activity of HK20

**Figure 9:**
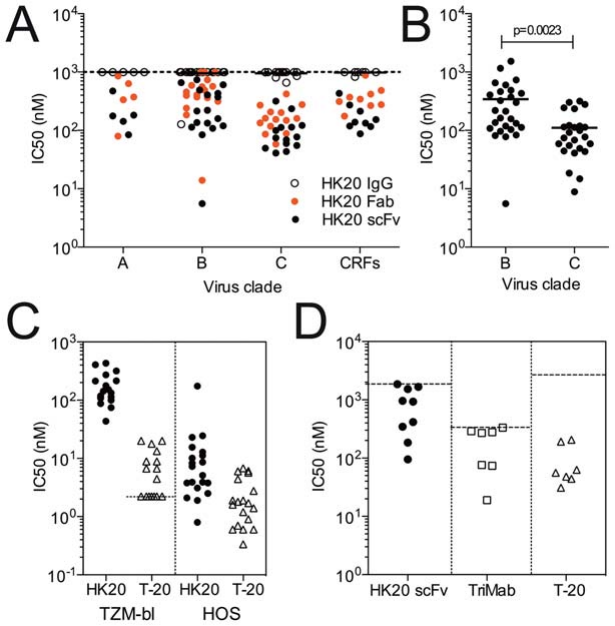
Size dependent neutralization breadth and potency of HK20 in different HIV-1 neutralization assays and comparison with T-20. (**A**) Neutralization of 45 HIV-1 isolates in TZM-bl cells by HK20 IgG (empty circles), Fab (red circles) and scFv (black circles). Shown are nM IC50 values. (**B**) Neutralization of 27 clade B and 25 clade C HIV-1 isolates in TZM-bl cells by HK20 scFv. Shown are nM IC50 values. Two-tailed P value was calculated with the unpaired t test. (**C**) HK20 scFv (black circles) and T-20 (empty triangles) were tested for their capacity to neutralize 20 HIV-1 isolates using either TZM-bl (left panel) or HOS (right panel) as target cells. Shown are nM IC50 values. The dotted line indicates the lowest concentration tested. (**D**) HK20 scFv (black circles), TriMab (2F5, 2G12 and b12, empty squares) and T-20 (empty triangles) were tested in a PBMC-based neutralization assay against a panel of 9 HIV-1 primary isolates from 5 different clades. Shown are nM IC90 values. The dotted lines indicate the highest concentration tested. Virus isolates used and detailed results are shown in Tables S1, S2, S3, S4, S5 in Supporting Information S1.

The HR1 region recognized by HK20 is only transiently exposed and has limited accessibility during the membrane fusion reaction [36] [37]. Our previous finding that HK20 Fab showed higher potency than IgG in an HOS-based assay [34] would be consistent with steric constraint in the accessibility of this epitope. We therefore tested whether further reducing the size of HK20 to single chain Fv (scFv) would increase neutralizing activity in the most demanding TZM-bl assay. HK20 IgG, Fab and scFv were compared in a TZM-b1-based assay against a panel of 45 Tier 1, 2 and 3 HIV-1 pseudoviruses (**Figure 9A** **and Table S2 in Supporting Information S1**). The HK20 Fab showed high breadth and potency, since it neutralized 43 out of the 45 viruses with IC50 values ranging from 14 to 1000 nM (**Figure 9A** **and Table S2 in Supporting Information S1**). HK20 scFv showed on average a 2–6 times higher potency as compared to the Fab and neutralized all 45 pseudoviruses with IC50 values ranging from 6 to 737 nM (**Figure 9A** **and Table S2 in Supporting Information S1**). Interestingly, HK20 scFv neutralized clade C isolates more potently than clade B viruses. This observation was further supported by testing HK20 scFv against a larger panel of clade B and C isolates (27 and 25 isolates, respectively, p = 0.0023) (**Figure 9B** **and Table S3 in Supporting Information S1**).

We then compared HK20 scFv with the T-20 peptide (Fuzeon) in both HOS and TZM-bl based assays using a panel of 20 isolates (**Figure 9C** **and Table S4 in Supporting Information S1**). HK20 scFv neutralized the same HIV-1 isolates with IC50 values 10–100 fold lower in HOS cells as compared to TZM-b1 cells (IC50 values ranging from 0.8 to 174 nM and 43 to 430 nM, respectively). T-20 neutralization was also more efficient in the HOS cells as compared to TZM-bl cells, with IC50 values ranging from <2.23 to 20 nM and 0.04 to 4.4 nM in TZM-bl and HOS assays, respectively. The breadth of HIV-1 neutralization by HK20 scFv is very high considering the high number of isolates tested and compares favorably with the reference broadly neutralizing mAbs b12 (27 out of 45), 2G12 (12 out of 45), 2F5 (22 out of 45), 4E10 (44 out of 45) and 447-52D (6 out of 45) (**Table S5 in Supporting Information S1**).

Given the limitations inherent to pseudoviruses and cell lines we compared HK20-scFv, T-20 and TriMab (a cocktail of the broadly neutralizing mAbs 2F5, 2G12 and b12) in a PBMC-based neutralization assays using nine primary replication-competent HIV-1 isolates representative of clades A, B, C, D and E (**Figure 9D** **and Table S6 in Supporting Information S1**). HK20 scFv neutralized 8 out of the 9 isolates tested with IC90 values ranging from 95 to 1667 nM. Interestingly, the two HIV-1 isolates neutralized more potently (i.e. Du174 and 92BR025) were clade C viruses.

In conclusion these results demonstrate that reduction of size dramatically increased HK20 scFv breadth and potency of viral neutralization in different assays, including PBMC-based assays.

## Discussion

In this study we present the crystal structure of the HR1-specific human mAb HK20 in complex with 5-Helix. HK20 binds to the same region recognized by the previously described mAb D5 [31], but differs significantly in the contact sites, in the angle of approach and shows a role for somatic mutations in affinity maturation. These aspects influence both potency and breadth of neutralization, which are higher for HK20 compared to D5 and depend on somatically mutated residues. In addition, we show that in case of HK20 the scFv is at least 15-fold more potent in neutralization than IgG, consistent with a limited accessibility to the target site.

The gp41 footprints of HK20 and D5 and the global structural principles employed by both antibodies are similar. CDR H2 and H3 loops contribute the main interactions in both cases whereas Phe 54 at the tip of CDR H2 points into a hydrophobic pocket that is occupied by gp41 HR2 residues in the post fusion conformation [15], [16], [31]. Notably, the same germline V gene family encodes the VH regions of HK20 and D5. While CDR2 H2 from D5 did not carry somatic mutations, HK20 CDR H2 is mutated and contains four amino acid changes which are required for high affinity binding. CDR H3 from HK20 and D5 follow distinct structural principles to contact the HR1 epitope, consistent with their different D and J gene usage. While HK20 CDR H2 contacts two HR1 helices, its CDR H3 contacts only one HR1 helix. In contrast, CDR H3 from D5 contacts two HR1 helices. As far as light chains are concerned, D5 uses all three light chain CDRs to interact with HR1 and HR2 and D5 CDR L3 contacts are important since mutation of CDR L3 Y94A leads to the loss of neutralization [31]. In contrast HK20 does not bind directly to HR2 and HK20 CDR L3 contributes only two hydrophobic contacts (Leu 94 and Asp 93). The minor role of the HK20 light chain is further underlined by our data showing that the light chain can be replaced by the germline sequence without loss of binding activity. In conclusion, HK20 employs primarily two CDR loops to contact gp41, while D5 utilizes all six CDRs, which might indicate that the interaction with gp41 is less rigid for HK20 as compared to D5. This might permit HK20 to engage with a higher degree of conformational flexibility, as suggested by normal mode analyses, which permit to study the vibrational and thermal properties of the complex [38]. Interestingly, the crystal structure reveals that HK20 and D5 Fabs approach the same HR1 epitope with different angles; while D5 binds almost orthogonal towards HR1, HK20 contacts 5-Helix in a ∼60° angle. It is conceivable that these different approaches may influence the accessibility to the target site.

Previous studies reported variable effects of size on viral neutralization by HR1 targeting molecules. For instance, the size of fusion proteins coupled to T-20 affected neutralization efficiency [36], while fusions to C34 had no effect [37]. D5 IgG and scFv showed comparable or slightly higher potency IC50 values [23], [39]. In contrast, HK20 Fab and scFv showed, on a molar basis, at least 15-fold higher potency as compared to the corresponding IgG. The fact that HK20 Fab and scFv efficiently neutralize all pseudoviruses tested indicates that the HR1 epitope is easily accessible for proteins of 50 kDa or less and that in the intact IgG the presence of the Fc and/or the second Fab arm obstructs epitope access. It is also possible that temporal constraints during the fusion reaction play an important role in explaining the lower activity of IgG over Fab/scFv, since the diffusion rate of IgG (4.9×10^−7^ cm^2^/sec) is slightly slower than that of Fab (7.4×10^−7^ cm^2^/sec) [40].

Both D5 and HK20 show similar kinetics of binding. Our SPR measurements indicated a K_d_ of 3.1 nM for D5, higher than the previously reported values of 0.26 nM [23] and 0.05 nM [31], which, however, have been obtained with 5-Helix as analyte and thus include contributions of HR2 binding. Thus the small differences in affinity are not necessarily the best correlate of viral neutralization, corroborating that neutralization is most likely critically dependent on accessibility to the HR1 target epitope and possibly other factors.

The conformational states of gp41 have been studied extensively and are attractive targets for fusion inhibitors and neutralizing antibodies due to the sequence conservation of gp41 [1], [13], [14], [32], [41], [42]. However, it is not clear whether this conserved site is sufficiently immunogenic to trigger a neutralizing antibody response *in vivo*. In addition, since HR1 is only transiently exposed during the fusion reaction, it is itself an unfavorable target for induction of a B cell response. On the other hand, since many Env complexes found on virions are non-functional, it is possible that the prolonged exposure of the HK20 epitope on such complexes induces HK20-like antibodies and favors their affinity maturation. Unlike D5, which was isolated from a phage library, HK20 was isolated from a memory B cell of an HIV-1 infected individual and was therefore selected *in vivo* in the course of HIV-1 infection. When reverted to the germline sequence HK20 still bound to the 5-Helix with 50-fold lower affinity but did not show neutralizing activity, indicating a critical role for somatic mutations to achieve high affinity binding and neutralization. Shuffling of Ig chains showed that mutations in the heavy but not in the light chain contributed to the increased binding affinity, a finding that is consistent with the structural data, which reveal only a minor role of CDR L3 for HR1 recognition.

In this study we used HK20 as a probe to quantify serum antibodies directed against the same site. Using an HK20 blocking assay we found that some HIV-1 infected individuals (20 out of 33) have variable levels of HK20-like antibodies with titers ranging from 1∶21 to 1∶528, which might include neutralizing and non-neutralizing antibodies. A recent study demonstrated that constructs containing HR1 and HR2 can be used to isolate by affinity chromatography from human immune sera antibodies with neutralizing activity in a PBMC-based assay [43]. However it remains to be determined which is the fraction of HR1-reactive antibodies endowed with neutralizing activity [24].

Different neutralization assays employing different target cells produce differences in neutralization breadth and potency with the result that no single assay is capable of detecting the entire spectrum of neutralizing activities [44]. Our findings demonstrate that antibodies that target HR1 are particularly sensitive to the target cell used. Indeed for both HK20 and D5 IgG we found much higher neutralization titers in the HOS-based as compared to the TZM-b1-based assay. It is possible that the high level of CD4 and CCR5 expression on TZM-b1 cells facilitates faster membrane fusion or might even cause a rapid internalization of the virus in the endosomal compartment thus limiting the time window available for antibodies to bind [45], [46]. It is also worth noting that HK20 potency is generally higher against clade C as compared to clade B viruses, which might be due to differences in the kinetics of virus entry.

The HR1 epitope recognized by HK20 is highly conserved (**Figure S3 in Supporting Information S1**) and only two positions tolerate amino acid exchanges. Notably, the JR-FL isolate has an Arg at position 564 rather than His or Gln which are found in >99.5% of the isolates and is only poorly neutralized by HK20 (IC50 174 nM on HOS cells). A similar finding was reported for mAb 8K8 [24]. Our HK20-gp41 structure demonstrates that His 564 is important to coordinate CDR H3 HR1 interaction, which can explain the poor neutralization of JR-FL.

T-20 is a peptide inhibitor that targets the gp41 fusion machinery [14]. HK20 scFv shows a neutralization breadth comparable to that of T-20 but on average a ∼3.4 fold lower potency on a molar basis in the HOS-based assay. Interestingly, the HK20 epitope does not overlap with the T-20 binding site and consequently we predict that HK20 will be able to neutralize T-20 escape mutants affecting the 547-GIV-549 region [47], [48]. Thus HK20 scFv represents a new tool to combat HIV-1 infection in general and specifically T-20-resistant viruses. Furthermore HK20 scFv or HK20 scFv mimetics might demonstrate an improved serum half life when compared to T-20 [49] or the next generation of peptide fusion inhibitors [50]. In addition, HK20 may synergize with HR2-specific antibodies or with small molecule entry inhibitors, as reported for mAb D5 in combination with mAb 2F5 [51].

Although HR1 is an attractive target for antibodies due to its high sequence conservation, an open question remains whether HK20-like antibodies are useful in the prevention of infection. While initial studies suggested that this might be a difficult goal [24], a recent study suggested that immunization with HR1 mimetics can induce relevant titers of HR-1 specific neutralizing antibodies in a species-dependent fashion [29].

In summary our structural and functional data illustrate the general principle that the conserved fusion machinery of gp41 can be targeted by antibodies produced in the course of HIV-1 infection. However, as demonstrated by HK20, the neutralizing activity may be severely limited by the accessibility of the epitope and by the size of the antibody. We propose that HK20 scFv, smaller engineered single domain HK20 versions or HK20 mimetics with oral bioavailability represent novel tools, complementary to T-20, to combat HIV-1 infection.

## Materials and Methods

### Protein expression and purification

The cDNA corresponding to 5-Helix [30] was synthesized (Geneart) and cloned into vector pETM-13 (EMBL, Heidelberg) and 5-Helix was expressed in *E. coli* strain BL21gold (DE3) (Invitrogen). Cells were grown to an OD_600nm_ of 0.7 and induced with 1 mM IPTG at 37°C. After 5 hours cells were harvested by centrifugation and lysed in buffer A (0.02 M Tris pH 8.0, 0.1 M NaCl). The soluble fraction was discarded and the pellet was resupended in buffer A supplemented with 0.2% octyl-β-*D*-glucopyranoside (Sigma) over night at 4°C. Solubilised proteins were separated on an anionic exchange column employing a gradient of buffer A and B (0.02 M Tris pH 8.0, 0.5 mM NaCl). Further purification was carried out by gel filtration on a Superdex 200 column (GE Healthcare) in buffer A. Gp41-HR1 is a modified version of a circular permutated gp41 where the C-helix precedes the N-helix in sequence and HR2 is truncated to expose the HR1 epitope [52]. Gp41-HR1 is fused N-terminally to a 30 amino acid triple stranded coil (pIQI) [53] to increase solubility. Gp41-HR1 was cloned into pETM11 and expressed in *E. coli* strain BL21gold (DE3) (Invitrogen). Cells were grown to an OD_600nm_ of 0.7 and induced with 1 mM IPTG at 37°C. After 3 hours cells were harvested by centrifugation and lysed in buffer A and purified on a Ni^2+^-NTA column. A final purification step included gel filtration on a S75 Superdex column in buffer A.

HK20 IgG was proteolysed for 4h at 37°C with immobilized papain (Roche) in buffer C (0.05 M Bis-Tris pH 6.3). Reaction was stopped with E-64 and filtering the solution through a 0.22um filter (Millipore). Proteolysed HK20 was dialyzed overnight at 4°C in buffer D (0.025 M sodium acetate pH 5.0) and Fab fragments were separated from Fc fragments by cationic exchange chromatography in buffer D. Further purification was achieved by passing the Fabs over a mono P chromatofocusing column (GE Healthcare) and a final step included gel filtration on a Superdex 200 column (GE Healthcare) in buffer A. HK20 Fabs were mixed with 5-Helix using a 0.5 M excess of HK20 Fabs. The complex was separated on a Superdex 75 column in buffer A and complex formation was confirmed by separation of the complex on a 12% SDS-Tris-Tricine gel and on a 10% native PAGE.

### Crystallisation, data collection and structure determination

HK20-5-Helix complex crystals were obtained by the vapour diffusion method in hanging drops mixing equal volumes of complex and reservoir solution (0.1 M Hepes pH 7.5, 0.2 M Ammonium sulphate, 25% PEG 3350 (w/v)). Crystal quality was improved and single crystals were obtained by microseeding of crystals into drops equilibrated with reservoir buffer (0.1 M Hepes pH 7.5, 0.2 M Ammonium sulphate, 21% PEG 3350 (w/v)).

The crystal was cryo-cooled at 100 K in reservoir buffer containing 25% (v/v) glycerol. A complete dataset was collected at the ESRF (Grenoble, France) microfocus beamline ID23-2. Data were processed and scaled with MOSFLM [54], and SCALA [55], [56]. The crystals belong to space group P2_1_ with unit cell dimensions of a = 75.95Å, b = 62.71Å, c = 76.61 and β = 97.70°. The structure was solved by molecular replacement using PHASER [57] and the D5-5-Helix complex structure as a search model (pdb ID 2CMR). The position of 5-Helix was first localized and the positions of heavy and light chains were successively determined. An initial model was build with ARP/WARP [58] and completed by several cycles of manual model building with COOT [59] and refinement with REFMAC [60] using data to 2.3 Å resolution (Table 1). The final model contains 5-Helix HR1 residues 543–584 (chain A), 541–581 (chain B), 543–582 (chain C) and HR2 residues 626–664 (chain D), 624–664 (chain E). The linker regions connecting HR1 and HR2 are disordered. HK20 residues include H chain residues 3–216 and L chain residues 1–214. Molecular graphics figures were generated with PyMOL (W. Delano; http://www.pymol.org). Co-ordinates and structure factures have been deposited in the Protein Data Bank with accession ID 2xra.

### Surface plasmon resonance

BIAcore measurements were performed with the Biacore X instrument (BIAcore. Inc.) at 25°C in running buffer (10 mM Tris pH 8.0, 100 mM NaCl, 0.005% surfactant P20, 50 µM NiCl). Ni^2+^ NTA chips were coated with gp41-HR1 to a target of 2000 response units (RU). The analyte mAbs D5 and HK20 (dialyzed o/n in running buffer) was passed over the chip surface at concentrations ranging from 1 nM to 50 nM for 160 seconds at a flow rate of 20 µl/min and dissociation was recorded during 5 minutes. The chip was regenerated with 20 µl of 35 mM EDTA at 50 µl/min. Binding kinetics were evaluated using the BiaEvaluation software package (BIAcore, Inc.) using a Langmuir model 1∶1 with no mass transfer.

### Viruses and mAbs

The clade B and C HIV-1 reference panels of Env clones [61] [62] and the SF162 clone were obtained from the NIH-AIDS Research and Reference Reagent Program (ARRRP). Other non-clade B isolates were provided by the Comprehensive Antibody Vaccine Immune Monitoring Consortium (CA-VIMC). HIV-1 subtype B clone JRFL was kindly provided by Dennis Burton (Scripps Institute, La Jolla, US). Pseudoviruses were produced by co-transfecting HEK293T/17 cells with the env expressing plasmids and the complementing viral-genome reporter gene vector, pNL4-3.Luc^+^.E^−^R^+^ (kindly provided by John R. Mascola, VRC, NIAID, NIH, US). TriMab was kindly provided by CFAR, NIBSC-HPA, UK. Virus supernatants Du174, CM244, 92UG024, RW9209, QH0692, VI191, 92BR025 and MN(P) were part of the NeutNet Virus Panel and distributed through the Programme EVA Centre for AIDS Reagents (CFAR) NIBSC-HPA, UK [44], and J213, a subtype B X4 virus is part of a pediatric virus isolation panel [63].

### Neutralization assays

TZM-b1 and HOS.CD4-R5 cells were obtained from the NIH AIDS Research and Reference Reagent program (ARRRP). A single cell infectivity assay was used to measure the neutralization of luciferase-encoding viruses pseudotyped with the HIV-1 Env proteins [64]. Both the HOS-CD4-R5 cell based assay and the TZM-b1 cell based assay were performed as described [34].

### PBMC-based virus titration and neutralization assay

Virus titrations (ID50) were performed in a PHA-activated PBMC culture, as previously described [65]. For the neutralization assay 10 to 50 Tissue Culture ID50 (TCID50) of virus were used, though a virus titration was always performed simultaneously with the neutralization assay to calculate the precise TCID50 of each test run. Each reagent was tested in duplicate, in 4 steps of 4-fold dilutions, against two 2-fold dilutions of virus. The protocol is available on the EUROPRISE website www.europrise.org, with a slight modification: cells were washed at day 3 by exchanging the culture supernatant with fresh culture medium. The culture supernatant was harvested at day 7 and tested with an *in house* HIV p24 antigen ELISA (Aalto Bio Reagents, Ireland).

### Analysis of antibody sequences and design of germline-like antibodies

The heavy and light chain nucleotide sequences were analyzed using the IMGT database. Germline-like sequences were determined by reverting mutations to the germline sequence while retaining the original CDR3 junctions and terminal deoxy-nucleotidyl transferase (TdT) N nucleotides.

### Gene synthesis and expression plasmid constructions

IgG and scFv DNAs corresponding to mature and germline-like HK20 and D5 heavy and light nucleotide sequences were synthesized by Genescript (Genescript, Piscatawy, NJ) and their accuracies were confirmed by sequencing. D5 VH and VK sequences were obtained through the Protein Data Bank (PDB accession code 2CMR). The scFv HK20 was designed to encode the VK gene followed by a (GGGGS) linker, the VH gene and a C-terminal His-tag. AgeI and HindIII restriction sites were added to 5′ and 3′ termini, respectively, during gene synthesis for cloning into the appropriate expression vectors. IgG1 expression vectors contained human IgG1 or Igκ constant regions (kindly provided by Michel Nussenzweig, Rockefeller University, New York, US). The His-tag was used subsequently for scFv purification.

### Recombinant antibody production and purification

Monoclonal antibodies were produced by transient transfection of suspension cultured 293 freestyle cells with PEI. Supernatants from transfected cells were collected after 7 days of culture. Recombinant IgG or scFv were affinity purified with Protein A or Ni^2+^ chromatography (GE Healthcare), respectively, according to the manufacturer's instructions, and finally desalted against PBS using a HiTrap FastDesalting column.

### Competition assay to detect HK20-like antibodies in patient sera

HK20 mAb was biotinylated using the EZ-Link NHS-PEO solid-phase biotinylation kit (Pierce). The competition between polyclonal serum antibodies and biotinylated mAb for binding to immobilized 5-Helix was measured by ELISA. Briefly, plasma samples were added to 5-Helix-coated plates at different dilutions. After 1 hour, biotinylated mAb was added at a concentration corresponding to 80% of the maximal OD level, and the mixture was incubated at room temperature for 1 hour. Plates were then washed and bound biotinylated mAb was detected using AP-labeled streptavidin (Jackson Immunoresearch). The percentage of inhibition was tested in duplicates and calculated as follows: (1−[(OD sample−OD neg ctr)/ (OD pos ctr−OD neg ctr)])×100. BD80 value was calculated by interpolation of curves fitted with a 4-parameter nonlinear regression.

### Sequence sources

The accession code of the env sequence of 5-Helix corresponds to the gp41 sequence of HXB2 (AAA76685; 5-Helix, 2CMR). The D5 heavy chain sequence (CH and VH) are deposited under 2CMR and the D5 light chain (CL and VL) under 2CMR in the NCBI protein database and SwissProt. The sequences of the HK20 heavy and light chains have been deposited with PDB ID code 2xar.

## Supporting Information

Supporting Information S1 Supplementary figures and tables
(2.22 MB PDF)
