# Supplementary material for: Crystal Structure and Size-Dependent Neutralization Properties of HK20, a Human Monoclonal Antibody Binding to the Highly Conserved Heptad Repeat 1 of gp41
Source: PLoS Pathog. 2010 Nov 18;6(11):e1001195. doi: 10.1371/journal.ppat.1001195 (PMC2987821; doi:10.1371/journal.ppat.1001195)
Supplement: Supporting Information S1 — Supplementary figures and tables (2.22 MB PDF) [file ppat.1001195.s001.pdf]

Supporting Information S1

Crystal structure and size-dependent neutralization properties of HK20, a human monoclonal antibody binding to the highly conserved heptad repeat 1 of gp41

Charles Sabin[1,10], Davide Corti[2,8,10], Victor Buzon[1,10], Mike S. Seaman[3], David Lutje Hulsik[1], Andreas Hinz[1], Fabrizia Vanzetta[4], Gloria Agatic[4], Chiara Silacci[2], Lara Mainetti[5], Gabriella Scarlatti[5], Federica Sallusto[2], Robin Weiss[6], Antonio Lanzavecchia[2,7] and Winfried Weissenhorn[1,9]

[1] Unit of Virus Host Cell Interactions (UVHCI) UMI 3265, Université Joseph Fourier-EMBL-CNRS, Grenoble, France

[2] Institute for Research in Biomedicine, Bellinzona, Switzerland

[3] Division of Viral Pathogenesis, Beth Israel Deaconess Medical Center, Boston, USA

[4] Humabs SAGL, Bellinzona, Switzerland

[5] Viral Evolution and Transmission Unit, Division of Immunology, Transplant and Infectious Diseases, San Raffaele Scientific Institute, Milan, Italy

[6] Division of Infection and Immunity, University College London, United Kingdom

[7] Institute of Microbiology, Swiss Federal Institute of Technology, Zurich, Switzerland

[8] Present address: Humabs SAGL, Bellinzona, Switzerland

[9] Corresponding author: e-mail: weissenhorn@embl.fr

[10] These authors contributed equally

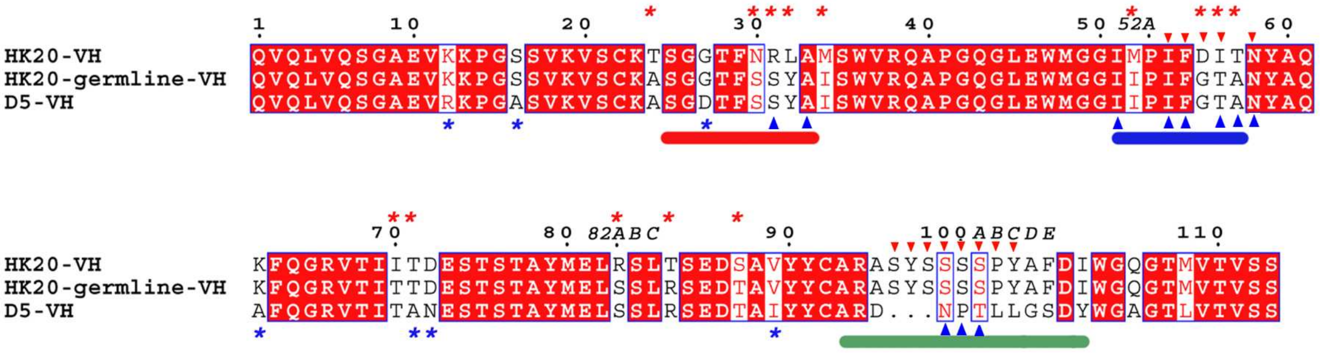

Figure S1. Sequence alignment of the heavy chain VH domains from HK20, the germline sequence of HK20 and of D5. CDR H regions 1-3 are indicated by red, blue and green bars. Note that HK20 uses the germline V_H gene VH1-69*05 and D5 the related gene VH1-69*01. Both antibodies use different D gene (D6-6*01 and D1-14*01) and J (J3*02 and J4*02) gene segments to encode V_H. Somatic mutations are indicated by asterisks. Note that somatic mutations within the V gene led to 13 amino acid exchanges in case of HK20 (red asterisks) and 7 changes in case of D5 (blue asterisks). Residues contacting gp41 are indicated as red triangles (HK20) and blue triangles (D5). The numbering corresponds to the HK20 sequence.

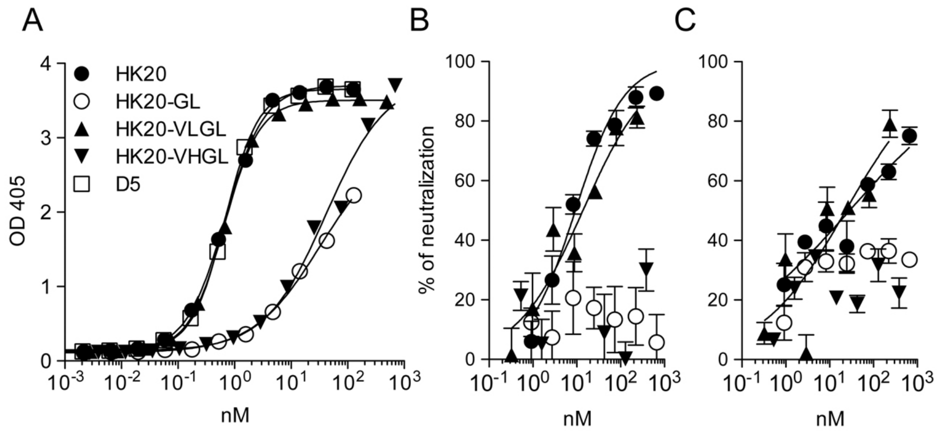

Figure S2. High affinity binding to 5-Helix and neutralizing activity of HK20 are the result of somatic hypermutation. (**A**) Binding of HK20 IgG variants and D5 to 5-Helix-coated ELISA plates. HK20-GL, VH and VL reverted to germline sequence; HK20-VLGL, VL reverted to germline; HK20-VHGL, VH reverted to germline. (**B-C**)

Neutralization of HIV-1 isolates Q769.d22 (B) and 96ZM651.2 (C) by HK20, HK20-GL, HK20-VLGL and HK20-VHGL in the HOS-cell based neutralization assay.

A

Sequence	%
QQHLLQLTVWGIKQLQ	41.37
QQHMLQLTVWGIKQLQ	30.35
QQHLLKLTVWGIKQLQ	9.33
QQHMLKLTVWGIKQLQ	2.11
QQHLLRLTVWGIKQLQ	2.01
QQQLLRLSVWGIRQLR	1.13
QQQLLKLTVWGIKQLQ	1.08
QQHLLRLSVWGIRQLR	0.98
QQHLLQLSIWGVKQLQ	0.82
QQHMLRLTVWGIKQLQ	0.77
QQHLLQLTVWGNKQLQ	0.72
QQQLLRLTVWGIKQLQ	0.67
QQHLLQLTVWGIKQLR	0.62
QQHILQLSVWGIKQLQ	0.52
QQRMLQLTVWGIKQLQ	0.52
Others	6.80

B

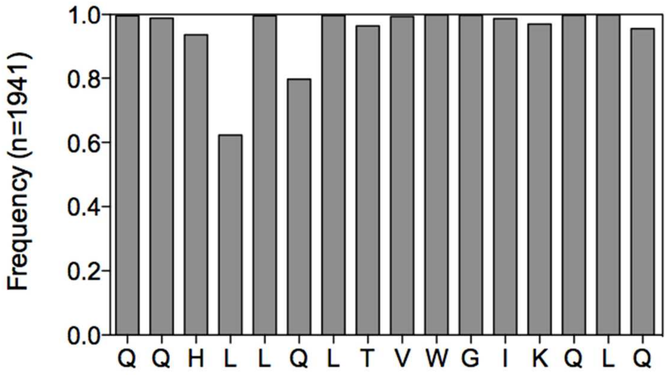

Figure S3 Amino acids critical for HK20 binding are highly conserved among HIV isolates. Analysis of the HK20 epitope region extracted from 1941 sequences obtained through the Los Alamos National Laboratory HIV sequence database. (**A**) Frequency of isolates encoding the indicated sequence in HR1. The gp41 contact residues with HK20 are indicated in red. (**B**) Frequency of the most representative amino acids among the 1941 analyzed sequences.

			IC50 (μg/ml)				IC50 (nM)			
			TZM-bl		HOS		TZM-bl		HOS	
Clade	Virus	Tier	HK20	D5	HK20	D5	HK20	D5	HK20	D5
A	Q461.e2	2	279	-	18	290	1858	-	121	1930
	Q769.d22	2	-	-	10	-	-	-	67	-
B	SF162	1A	-	-	11	48	-	-	73	320
	BaL.26	1B	-	-	27	245	-	-	180	1633
	SS1196.1	1B	-	-	30	-	-	-	200	-
	AC10.0.29	2	-	-	16	141	-	-	107	940
	RHPA4259.7	2	-	-	176	-	-	-	1173	-
C	Du151.2	2	-	-	17	90	-	-	113	600
	ZM53M.PB12	2	-	-	6	71	-	-	40	473
	CAP210.2.00.E8	2	-	-	6	71	-	-	40	473
	96ZM651.2	2	200	-	1	52	1336	-	7	347
	MW965.26	1A	63	247	6	19	421	1649	40	127
AD	Q168.a2	2	-	-	28	-	-	-	187	-
AG	T257-31	3	-	-	9	-	-	-	60	-
	263-8	2	-	-	34	-	-	-	227	-
BC	CH064.20	2	260	-	6	25	1733	-	40	167
	CH110.2	2	-	-	15	-	-	-	100	-
	CH181.12	2	-	-	37	229	-	-	250	1527
	MuLV		-	-	-	-	-	-	-	-

Table S1 Comparative neutralization of HK20 IgG and D5 IgG in both TZM-bl and HOS-based neutralization assays. Neutralization of 18 HIV-1 isolates in TZM-bl cells and HOS cells by HK20 IgG and D5 IgG. HK20 and D5 were tested for their capacity to neutralize the same 18 HIV-1 pseudoviruses representing 6 different clades. Shown are IC50 values in µg/ml and nM. -, IC50 >2000 nM (>300 µg/ml). MuLV neutralization was included as negative control.

	Env clone	IC50 (μg/ml) IgG	IC50 (μg/ml) Fab	IC50 (μg/ml) scFv	IC50 (nM) IgG	IC50 (nM) Fab	IC50 (nM) scFv
A	Q461.e2	-	18.6	4	-	372	143
	Q769.d22	-	31.7	5	-	634	177
	MS208.A1	150	4	2	1000	80	84
	92RW020.2	-	16.8	5	-	336	183
	Q259.d2.17	-	42.8	13	-	856	474
B	SF162	-	29.5	3	-	590	109
	BaL.26	-	22.7	6	-	454	214
	SS1196.1	-	28.1	12	-	562	431
	AC10.0.29	-	20.3	3	-	406	113
	RHPA4259.7	-	19.9	11	-	398	406
	TRO.11	-	15.8	6	-	316	221
	MN-3	19	0.7	0	127	14	6
	WITO4160.33	-	12	2	-	240	84
	PVO.4	-	36.6	20	-	732	737
	6535.3	-	-	4	-	-	133
	SC42261.8	-	9.2	3	-	184	120
	QH0692.42	-	28.5	18	-	570	656
	BX08.16	-	-	16	-	-	604
	THRO4153.18	-	50	13	-	1000	490
	REJO4541.67	-	18.6	4	-	372	159
	CAAN5342.A2	-	24.4	4	-	488	137
	TRJO4551.58	-	18.1	10	-	362	373
C	Du151.2	-	13.8	9	-	276	317
	ZM53M.PB12	-	4.3	3	-	86	99
	CAP210.2.00.E8	-	6.7	2	-	134	75
	96ZM651.2	128	7.4	3	856	148	114
	Du422.1	-	4.4	1	-	88	55
	CAP45.2.00.E8	-	13.5	2	-	270	77
	Du156.12	121	5.9	2	805	118	59
	Du172.17	149	7.7	1	991	154	49
	ZM214M.PL15	-	8	1	-	160	43
	ZM249M.PL1	-	8.1	2	-	162	69
	ZM197MPB7	-	21.1	6	-	422	236
	ZM233M.PB6	99	2.9	1	660	58	41
	ZM109F.PB4	-	10.1	3	-	202	122
	ZM135M.PL10a	-	10.3	3	-	206	112
AD	Q168.a2	-	18.5	6	-	370	213
AG	T257-31	-	20.9	7	-	418	273
	263-8	-	24.2	4	-	484	154
	211-9	-	13.8	3	-	276	117
	271-11	-	43.9	12	-	878	431
BC	CH064.20	125	15.6	3	831	312	118
	CH110.2	-	8.8	2	-	176	88
	CH181.12	-	13.3	4	-	266	134
	CH111.18	-	17.1	4	-	342	138
	MuLV	-	-	-	-	-	-

Table S2. Size dependent neutralization breadth and potency of HK20 in the TZM-bl based neutralization assay. HK20 IgG, Fab and scFv were tested for their capacity to neutralize 45 HIV-1 pseudoviruses representing 6 different clades including Tier-1, Tier-2 and Tier-3 isolates using TZM-bl as target cells. Shown are IC50 values expressed in µg/ml and nM. -, IC50 >1000 nM.

Clade	Virus	µg/ml IC50	µg/ml IC90	nM IC50	nM IC90
B	SF162.LS	3	37	109	1363
	BaL.26	6	41	213	1502
	SS1196.1	12	47	430	1727
	AC10.0.29	3	22	113	831
	RHPA4259.7	11	>50	406	-
	MN-3	0	2	6	64
	Bx08.16	16	50	602	1852
	6535.3	4	>50	133	-
	QH0692.42	18	>50	655	-
	SC422661.8	3	23	119	836
	PVO.4	20	>50	735	-
	TRO.11	6	42	220	1556
	THRO4156.18	13	>50	489	-
	REJO4541.67	4	>50	158	-
	TRJO4551.58	10	45	372	1673
	WITO4160.33	2	16	84	577
	CAAN5342.A2	4	22	136	814
	WEAU-d15.410.787	2	8	81	300
	BB1006-11.C2.1601	31	-	1163	-
	BB1054-07.TC4.1499	4	-	163	-
	BB1056-10.TA11.1826	3	45	96	1667
	BB101211.TC21	3	33	104	1222
	H022.7	8	-	300	-
	H030.4	9	44	326	1622
	H077.31	2	43	78	1581
	H035.18	13	-	463	-
	H061.14	41	-	1530	-
C	Du151.2	9	35	317	1285
	ZM53M.PB12	3	15	99	555
	CAP210.2.00.E8	2	16	74	596
	96ZM651.02	3	24	113	889
	MW965.26	0	2	9	77
	Du156.12	2	18	59	649
	Du172.17	1	10	49	373
	Du422.1	1	12	55	462
	ZM197M.PB7	6	36	236	1343
	ZM214M.PL15	1	26	43	950
	ZM233M.PB6	1	5	41	199
	ZM249M.PL1	2	15	69	572
	ZM109F.PB4	3	24	121	886
	ZM135M.PL10a	3	13	112	497
	CAP45.2.00.E8	2	43	77	1584
	16845-2.22	8	40	278	1485
	16936-2.21	2	5	59	196
	25711-2.4	8	28	304	1048
	0013095-2.11	1	3	19	122
	001428-2.42	3	20	115	744
	0921.v2.c14	3	20	93	726
	0041.v3.c18	1	6	44	226
	3873.v1.c24	3	50	119	1852
	6322.v4.c1	7	34	244	1274
	0077.v1.c16	0	10	15	385

Table S3. HK20 scFv neutralizes more potently clade C viruses. Neutralization of 27 clade B and 25 clade C HIV-1 isolates in TZM-bl cells by HK20 scFv. Shown are IC50 and IC90 values expressed in µg/ml and nM. -, IC50 >1850 nM.

Clade	Virus	IC50 (nM) HK20 scFv HOS	IC50 (nM) HK20 scFv TZMbl	IC50 (nM) T20 HOS	IC50 (nM) T20 TZMbl
A	Q461.e2	5.1	143.29	1.6	6.68
	Q769.d22	2.5	176.7	1.7	20.04
B	SF162	3.9	108.76	6.8	20.04
	BaL.26	24.5	213.82	1.4	6.68
	SS1196.1	11.2	430.97	0.9	4.45
	AC10.0.29	3.9	113.22	1.2	6.68
	RHPA4259.7	23	406.47	1.9	8.9
	JR-FL	174	nd	1.9	nd
C	Du151.2	8.3	317.38	0.6	<2.23
	ZM53M.PB12	3.8	99.48	6.1	<2.23
	CAP210.2.00.E8	3.1	74.61	6	13.36
	96ZM651.2	0.8	113.59	0.6	8.9
	MW965.26	2.1	nd	0.6	nd
	ZM214M.PL15	3.791	43.43	4.425	17.81
AD	Q168.a2	15.5	213.07	5.7	8.9
AG	T257-31	10.15	273.21	2.76	<2.23
	263-8	12.8	153.68	0.04	<2.23
BC	CH064.20	1.9	118.42	0.7	<2.23
	CH110.2	7.265	87.61	0.3349	<2.23
	CH181.12	8.6	133.64	1.8	<2.23

Table S4. Neutralization of 20 HIV-1 isolates in HOS and TZM-bl cells by HK20 scFv and T20. HK20 and T20 were tested for their capacity to neutralize the same 20 HIV-1 pseudoviruses representing 6 different clades using either HOS-based or TZM-bl as target cells. Shown are nM IC50 values. nd, not determined.

					IC50 (µg/ml)		IC50 (nM)		IC50 (µg/ml)					IC50 (nM)				
	Env clone	Tier	Year	Location	HK20 scFv	T-20	HK20 scFv	T-20	b12	2G12	2F5	4E10	447-52D	b12	2G12	2F5	4E10	447-52D
	MS208.A1	1	?	Montserrat	50.00	0.02	1856.0	4.45	1.0	-*	0.80	0.70	nd	6.7	-	5.3	4.7	-
	92RW020.2	1	1992	Rwanda	4.93	<0.01	183.01	<2.23	19.0	-*	5.50	4.80	nd	126.7	-	36.7	32.0	-
A	Q769.d22	2	?	Kenya	4.76	0.09	176.70	20.04	50.00	-	0.50	0.40	-	333.3	-	3.3	2.7	-
	Q461.e2	2	?	Kenya	3.86	0.03	143.29	6.68	-	-	4.10	2.00	-	-	-	27.3	13.3	-
	Q259.d2.17	2	?	Kenya	12.77	<0.01	474.03	<2.23	-	-	7.10	8.50	-	-	-	47.3	56.7	-
	BaL.26	1B	1985	USA	5.76	0.03	213.82	6.68	0.20	0.90	0.80	0.70	0.04	1.3	6.0	5.3	4.7	0.3
	TRO.11	2	1995	Italy	5.95	0.05	220.87	11.13	-	0.40	-	0.30	-	-	2.7	-	2.0	-
	SF162	1A	?	San Francisco	2.93	0.09	108.76	20.04	0.01	0.70	0.90	1.60	<0.02	0.1	4.7	6.0	10.7	-
	MN-3	1A	?	USA	0.15	<0.01	5.57	<2.23	0.1	nd	nd	nd	<0.02	0.7	-	-	-	-
	WITO4160.33	2	2000	Alabama	2.26	<0.01	83.89	<2.23	3.1	1.10	0.60	0.30	-	20.7	7.3	4.0	2.0	-
	PVO.4	3	1996	Italy	19.85	0.30	736.85	66.79	-	1.20	-	6.50	-	-	8.0	-	43.3	-
	6535.3	2	1995	Washington D.C.	3.58	<0.01	132.89	<2.23	1.4	2.00	1.90	0.20	0.10	9.3	13.3	12.7	1.3	0.7
	SC42261.8	2	1995	Trinidad	3.22	0.03	119.53	6.68	0.2	2.10	0.70	0.90	-	1.3	14.0	4.7	6.0	-
B	QH0692.42	2	1994	Trinidad	17.68	0.97	656.30	215.94	0.3	2.80	1.00	1.40	39.30	2.0	18.7	6.7	9.3	262.0
	BX08.16	1B	?	France	16.26	0.02	603.59	4.45	4.2	5.40	2.60	2.40	0.10	28.0	36.0	17.3	16.0	0.7
	SS1196.1	1B	1997	USA	11.61	0.02	430.97	4.45	0.3	10.80	21.80	0.40	0.40	2.0	72.0	145.3	2.7	2.7
	RHPA4259.7	2	2000	Tennessee	10.95	0.04	406.47	8.90	0.1	-	12.00	6.90	48.90	0.7	-	80.0	46.0	326.0
	THRO4153.18	2	2000	Alabama	13.21	0.12	490.37	26.71	0.5	-	-	0.30	-	3.3	-	-	2.0	-
	REJO4541.67	2	2001	Alabama	4.27	0.06	158.51	13.36	0.7	-	0.60	0.70	-	4.7	-	4.0	4.7	-
	AC10.0.29	2	1998	Massachusetts	3.05	0.03	113.22	6.68	1.9	-	1.30	0.30	-	12.7	-	8.7	2.0	-
	CAAN5342.A2	2	2004	Alabama	3.68	0.07	136.60	15.58	-	-	3.60	2.70	-	-	-	24.0	18.0	-
	TRJO4551.58	3	2001	Alabama	10.05	<0.01	373.07	<2.23	-	-	-	4.50	-	-	-	-	30.0	-
	Du422.1	2	1998	Durban	1.49	<0.01	55.31	<2.23	0.2	-	-	0.70	-	1.3	-	-	4.7	-
	CAP45.2.00.E8	2	2005	Durban	2.08	0.03	77.21	6.68	0.7	-	-	2.60	-	4.7	-	-	17.3	-
	Du156.12	2	1999	Durban	1.60	<0.01	59.39	<2.23	0.8	-	-	0.20	-	5.3	-	-	1.3	-
	Du172.17	2	1998	Durban	1.33	<0.01	49.37	<2.23	1.0	-	-	0.30	-	6.7	-	-	2.0	-
	Du151.2	2	1998	Durban	8.55	<0.01	317.38	<2.23	1.4	-	-	0.80	-	9.3	-	-	5.3	-
	ZM214M.PL15	2	2003	Lusaka	1.17	0.08	43.43	17.81	3.0	-	-	4.00	-	20.0	-	-	26.7	-
C	ZM249M.PL1	2	2003	Lusaka	1.85	<0.01	68.67	<2.23	3.2	-	-	2.10	-	21.3	-	-	14.0	-
	ZM197MPB7	2	2002	Lusaka	6.36	0.01	236.09	2.23	19.9	-	12.30	0.50	-	132.7	-	82.0	3.3	-
	CAP210.2.00.E8	2	2005	Durban	2.01	0.06	74.61	13.36	20.4	-	-	1.20	-	136.0	-	-	8.0	-
	ZM53M.PB12	2	2000	Lusaka	2.68	0.01	99.48	2.23	25.9	-	-	7.00	-	172.7	-	-	46.7	-
	ZM233M.PB6	2	2002	Lusaka	1.10	<0.01	40.83	<2.23	-	-	-	1.20	-	-	-	-	8.0	-
	ZM109F.PB4	2	2000	Lusaka	3.28	<0.01	121.76	<2.23	-	-	-	0.60	-	-	-	-	4.0	-
	96ZM651.2	2	1996	Zambia	3.06	0.04	113.59	8.90	-	nd	nd	nd	nd	-	-	-	-	-
	ZM135M.PL10a	2	1998	Lusaka	3.03	0.09	112.48	20.04	-	-	-	0.60	-	-	-	-	4.0	-
AD	Q168.a2	2	1995	Kenya	5.74	0.04	213.07	8.90	-	-	3.20	2.30	-	-	-	21.3	15.3	-
	263-8	2	?	W. Afr	4.14	<0.01	153.68	<2.23	-	15.00	-	1.40	-	-	100.0	-	9.3	-
AG	211-9	2	?	Cameroon	3.14	0.04	116.56	8.90	-	38.30	4.70	5.90	-	-	255.3	31.3	39.3	-
	T257-31	3	?	W. Afr.	7.36	<0.01	273.21	<2.23	-	-	1.00	1.20	-	-	-	6.7	8.0	-
	271-11	2	?	Cameroon	11.62	<0.01	431.34	<2.23	38.7	-	7.00	2.10	-	258.0	-	46.7	14.0	-
	CH181.12	2	?	Beijing	3.60	<0.01	133.64	<2.23	2.5	-*	-*	0.80	-	16.7	-	-	5.3	-
BC	CH110.2	2	?	China	2.36	<0.01	87.61	<2.23	10.8	-*	-*	0.10	-	72.0	-	-	0.7	-
	CH064.20	2	?	Beijing	3.19	<0.01	118.42	<2.23	-	-*	-*	0.40	-	-	-	-	2.7	-
	CH111.18	2	?	China	3.73	<0.01	138.46	<2.23	-	-*	-*	1.50	-	-	-	-	10.0	-
	MuLV				-	-	-	-	-	-	-	-	-	-	-	-	-	-

Table S5 Neutralization of 45 HIV-1 isolates in TZM-bl cells by HK20 scFv. HK20 scFv were tested in comparison to T-20, b12, 2G12, 2F5, 4E10 and 447-52D to neutralize 45 HIV-1 pseudoviruses representing 5 different clades representing Tier-1 and Tier-2 isolates using TZM-bl as target cells. Shown are IC50 values expressed in µg/ml and nM. Results shown here for HK20 scFv and T-20 were also used in Figure 9A-B-C and Table S2. -, IC50 >333 nM (50 µg/ml) in the case of b12, 2G12, 2F5, 4E10; -, IC50>5100 nM (25 µg/ml) in the case of T-20; -, IC50>1850 nM (50 µg/ml) in the case of HK20 scFv. * mAbs tested starting from 166 nM (25 µg/ml). nd, not determined.

Clade	Virus	IC50 (nM) HK20 scFv	IC50 (nM) Trimab	IC50 (nM) T20	IC90 (nM) HK20 scFv	IC90 (nM) Trimab	IC90 (nM) T20
A	VI 191	932	88	32	1667	271	56
	92RW009	252	152	<16	956	290	44
B	QH0692	655	<5	78	1536	19	207
	MN(P)	108	nd	39	932	nd	63
	J213	>1850	136	35	>1850	279	192
C	Du174	30	46	nd	95	76	nd
	92BR025	<29	19	<16	184	>333	29
D	92UG024	243	44	16	416	74	48
E	CM244	<111	nd	nd	346	nd	nd

Table S6. Neutralization of 9 HIV-1 infectious isolates in a PBMC-based neutralization assay. HK20 scFv, Trimab (2F5, 2G12 and b12) and T-20 were tested in a PBMC-based neutralization assay against a panel of 9 HIV-1 infectious isolates from 5 different clades. Shown are nM IC50 and IC90 values. -, IC50>1850 nM (50 µg/ml) in the case of HK20 scFv. -, IC50 >333 nM (50 µg/ml) in the case of Trimab.
